# Crystal structures of ethyl {2-[4-(4-iso­propyl­phen­yl)thia­zol-2-yl]phen­yl}carbamate and ethyl {2-[4-(3-nitro­phen­yl)thia­zol-2-yl]phen­yl}carbamate

**DOI:** 10.1107/S2056989016013104

**Published:** 2016-08-19

**Authors:** Elena V. Sukhonosova, Sergey A. Sokov, Gennady I. Ostapenko, Alexander S. Bunev, Pavel V. Dorovatovskii, Yan V. Zubavichus, Victor N. Khrustalev

**Affiliations:** a Laboratory of Functional Heterocyclic Compounds, Togliatti State University, 14 Belorusskaya St., Togliatti 445020, Russian Federation; b National Research Centre "Kurchatov Institute", 1 Acad. Kurchatov Sq., Moscow 123182, Russian Federation; c Inorganic Chemistry Department, Peoples’ Friendship University of Russia, 6 Miklukho-Maklay St., Moscow 117198, Russian Federation; d X-Ray Structural Centre, A.N. Nesmeyanov Institute of Organoelement Compounds, Russian Academy of Sciences, 28 Vavilov St., B–334, Moscow 119991, Russian Federation

**Keywords:** crystal structure, Thio­sporine B analogs, thia­zoles, carbamates, hydrogen bonding

## Abstract

The title compounds, C_21_H_22_N_2_O_2_S (I) and C_18_H_15_N_3_O_4_S (II), are structural analogs of the alkaloid Thio­sporine B. Both mol­ecules adopt a near-planar V-shaped conformation, which is consolidated by intra­molecular N—H⋯N and C—H⋯O hydrogen bonds. The crystal structure of (I) consists of mlecular stacks along the *a* axis, in which the mol­ecules are linked to each other by π(S)⋯π(C) inter­actions. In the crystal of (II), mol­ecules are linked into chains by C—H⋯O hydrogen bonds and the chains are cross-linked into (100) sheets by π–π stacking inter­actions.

## Abstract 2

Two new thia­zole derivatives – the structural analogs of the alkaloid Thio­sporine B – were studied by X-ray diffraction.

## Chemical context   

Marine actinomycetes are prolific producers of biologically active natural products. This unique habitat has led to the abundant chemical diversity of metabolites that provides a foundation for the discovery of promising drug lead compounds. Among all known marine microbial secondary metabolites, over half were produced by actinomycetes (Fenical & Jensen, 2006 ▸; Lam *et al.*, 2006 ▸; Fu *et al.*, 2011 ▸). From this resource, more than 400 new active secondary metabolites have been isolated (Bérdy, 2005 ▸; Bull & Stach, 2007 ▸; Molinski *et al.*, 2009 ▸). Some of them represented by abyssomycin C (Bister *et al.*, 2004 ▸), diazepinomicin (Charan *et al.*, 2004 ▸), salinosporamide A (Feling *et al.*, 2003 ▸) and the marinomycins (Kwon *et al.*, 2006 ▸) are potent anti­biotics and possess novel structures. A comparatively large class of natural compounds possessing biological activity contains imidazole, thia­zole, or oxazole moieties. Studies of biological activity (Zabriskie *et al.*, 1990 ▸; Carroll *et al.*, 1996 ▸; Taori *et al.*, 2008 ▸) as well as a total synthesis of thia­zoles containing alkaloids isolated from marine microorganisms are very important directions. In many cases, the substances mentioned above have promising anti­tumor (Luesch *et al.*, 2001 ▸) and anti­bacterial (Shimanaka *et al.*, 1994 ▸; Yun *et al.*, 1994 ▸) activities.

In this paper we report a synthetic approach to the preparation of new thia­zole derivatives (I) and (II) containing aryl fragments – the structural analogs of alkaloid Thio­sporine B (Fu & MacMillan, 2015 ▸) – and their investigation by single crystal X-ray diffraction.
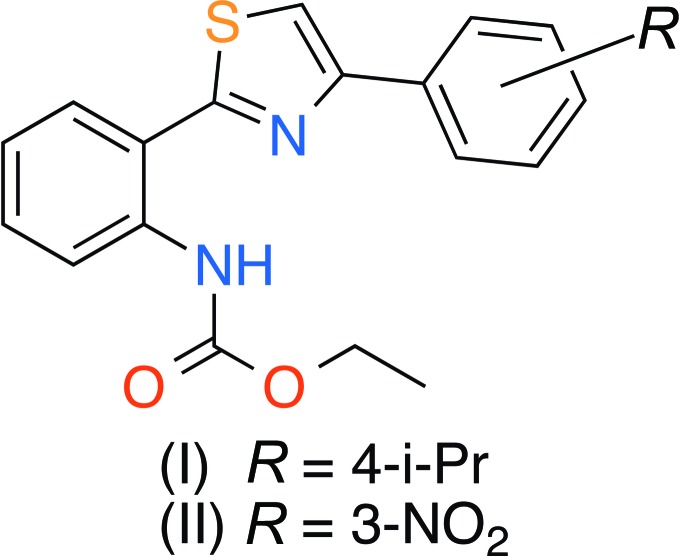


## Structural commentary   

**Figure 1:**
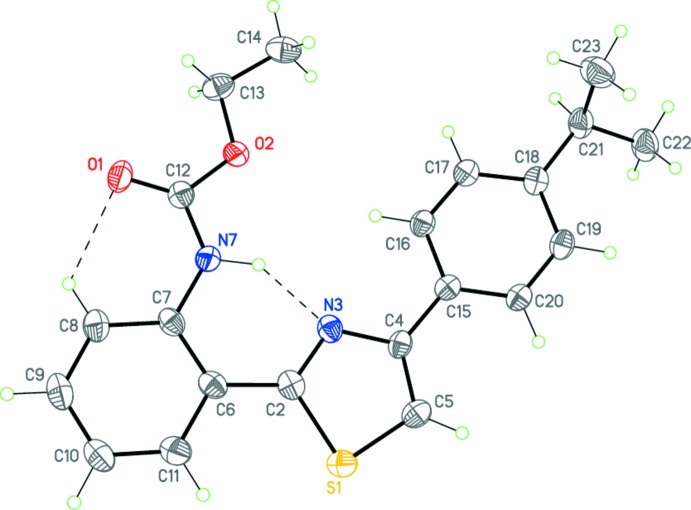
The mol­ecular structure of (I). Displacement ellipsoids are shown at the 50% probability level. Dashed lines indicate the intra­molecular hydrogen bonds. H atoms are presented as small spheres of arbitrary radius.

Compounds (I), C_21_H_22_N_2_O_2_S, and (II), C_18_H_15_N_3_O_4_S, have very similar mol­ecular geometries (Figs. 1 ▸ and 2 ▸), allowing for the different substituents on the benzene rings. Both mol­ecules adopt a near-planar V-shaped conformation, which is consolidated by intra­molecular N7—H7⋯N3 and C8—H8⋯O1 hydrogen bonds (Tables 1 ▸ and 2 ▸, Figs. 1 ▸ and 2 ▸) as well as an inter­molecular π–π inter­actions (see Section 3 below). There exists a small twist of 10.27 (15)° between the central thia­zole and 4-benzene rings in (I) only. Surprisingly, the ethyl (phen­yl)carbamate substituents (with the exception of some hydrogen atoms of the ethyl fragment) are perfectly coplanar with the thia­zole ring in both mol­ecules.

The bond-length distributions within the thia­zole rings of (I) and (II) are almost identical, clearly indicating that some degree of delocalization is present. These values are in good agreement with those observed in related structures (Garden *et al.*, 2007 ▸; Sen *et al.*, 2013 ▸; Bunev *et al.*, 2014 ▸; Mague *et al.*, 2014 ▸; Moreno-Fuquen *et al.*, 2015 ▸; AaminaNaaz *et al.*, 2015 ▸). The C—S—C angle in (I) [89.70 (12)°] and (II) [89.94 (12)°] is also very close to that in the previously reported analogous structures [89.0 (2)–90.3 (5)°; Nayak *et al.*, 2009 ▸; Hua *et al.*, 2014 ▸).

## Supra­molecular features   

Although the similarity of the mol­ecular geometries and types of intra­molecular inter­actions might lead to similar packing motifs, this is not found in the case of (I) and (II). The inter­molecular inter­actions, namely, π–π inter­actions and C—H⋯O hydrogen bonding, combined in a different way, give rise to various packing networks.

**Figure 3:**
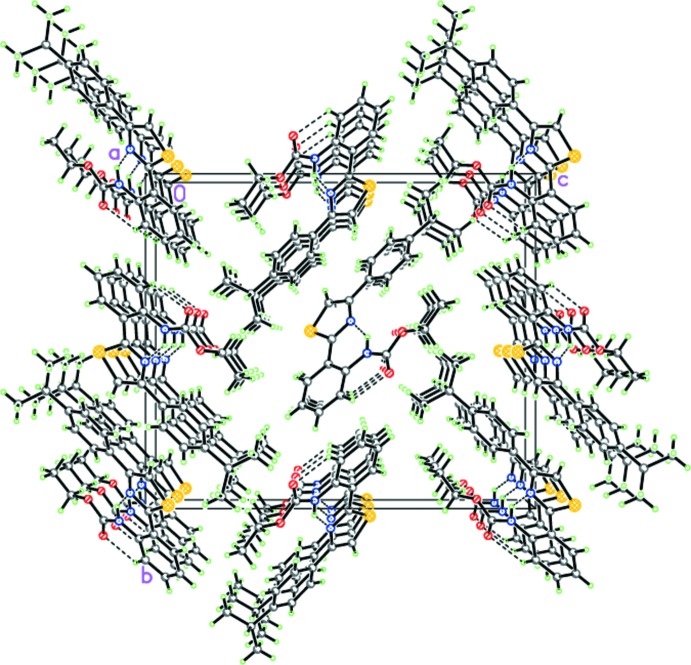
The crystal structure of (I). Dashed lines indicate the intra­molecular N—H⋯N and C—H⋯O hydrogen bonds.

**Figure 4:**
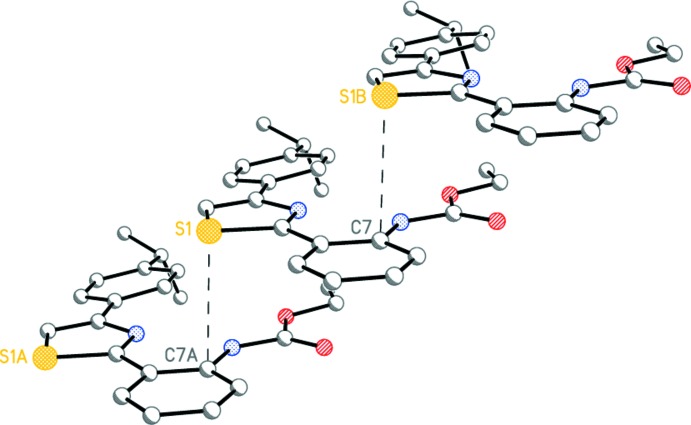
A fragment of the stack in (I). Dashed lines indicate the inter­molecular S⋯C inter­actions within the stack.

In (I), the crystal packing consists of stacks along the *a* axis (Fig. 3 ▸), in which the mol­ecules are linked to each other by π(S1)⋯π(C7) [1 + *x*, *y*, *z*] inter­actions at distances of 3.463 (3) Å (Fig. 4 ▸). No other directional inter­molecular inter­actions are observed in (I).

**Figure 5:**
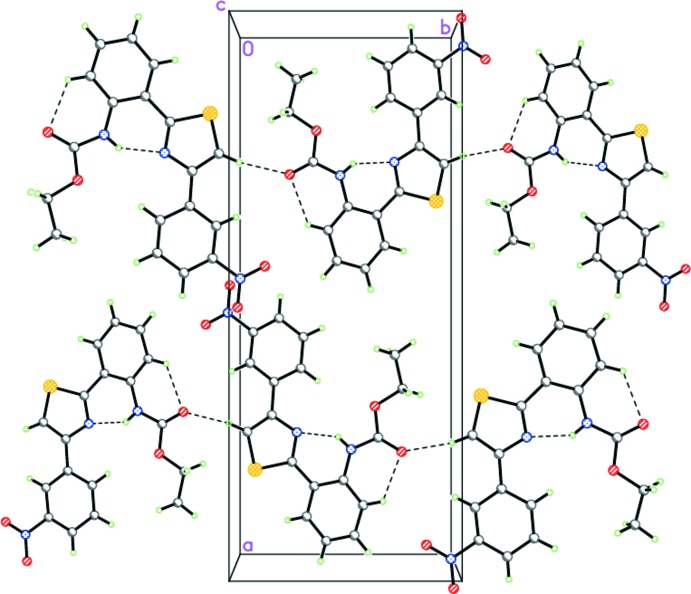
The hydrogen-bonded chains of (II). Dashed lines indicate the intra­molecular N—H⋯N and C—H⋯O and inter­molecular C—H⋯O hydrogen bonds.

**Figure 6:**
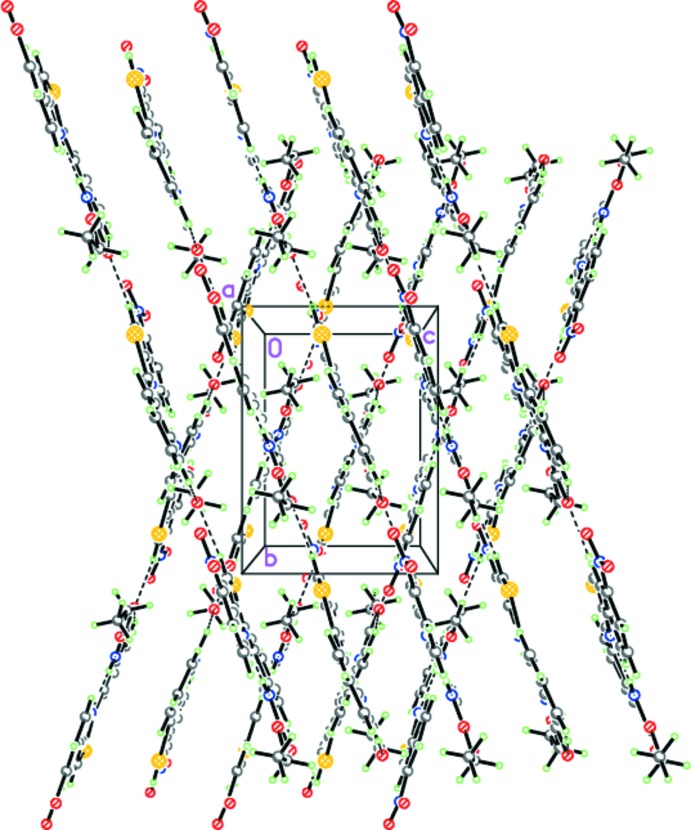
Crystal structure of (II) demonstrating the mutual arrangement of the hydrogen-bonded chains. Dashed lines indicate the intra­molecular N—H⋯N and C—H⋯O and inter­molecular C—H⋯O hydrogen bonds.

The situation in the case of (II) is quite different. The mol­ecules of (II) form chains *via* C5—H5⋯O1(−*x* + 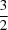, *y* − 1, *z* − 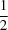) hydrogen bonds (Table 1 ▸, Fig. 5 ▸). It should be pointed out that the mol­ecules within the chains are coplanar, forming a ribbon-like motif. Further, the ribbons are packed in layers parallel to (100) *via* π–π stacking inter­actions (Fig. 6 ▸). The distance between the ribbons in the layers is 3.216 (3) Å. Importantly, the ribbons of adjacent layers are not parallel to each other, but disposed at an inter­plane angle of 39.91 (2)° (Fig. 6 ▸). Thus, the crystal of (II) comprises alternating layers, in which mol­ecules are arranged in a different manner.

## Synthesis and crystallization   

**Figure 7:**
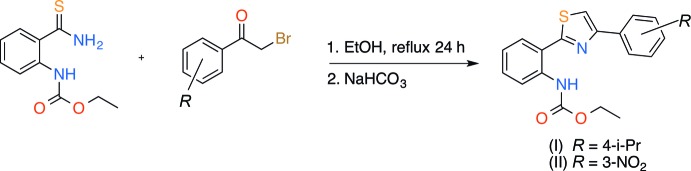
Synthesis of the title thia­zoles (I) and (II).

A solution of ethyl (2-carbamo­thio­ylphen­yl)carbamate (2.24 g, 10 mmol) and the appropriately substituted phenacyl bromide (10 mmol) in 95% EtOH (50 ml) was heated for 12 h under reflux. After cooling to room temperature, the solution was basified with saturated NaHCO_3_ solution to yield the expected product (I) or (II) (Fig. 7 ▸). The reaction mixture was filtered and the isolated solid was washed with water and dried *in vacuo*. The compounds were isolated as pale-yellow crystalline solids in 51% and 74% yield for the *i*-propyl (I) and nitro (II) derivatives, respectively. Single crystals of the products were obtained by slow crystallization from *N*,*N*-di­methyl­formamide solution.

**Spectroscopic and physical data for (I):** M.p. 379-381 K. FT–IR (ν_max_, cm^−1^): 3090, 1982, 1725, 1603, 1544, 1487, 1312, 1240, 1071. ^1^H NMR (600 MHz, DMSO-*d*_6_, 304 K): *δ* = 1.25 (*d*, 6H, *J* = 6.9), 1.33 (*t*, 3H, *J* = 7.1), 2.96 (*h*, 1H, *J* = 7.2), 4.21 (*q*, 2H, *J* = 7.1), 7.19 (*t*, 1H, *J* = 7.6), 7.37 (*d*, 2H, *J* = 8.2), 7.50 (*t*, 1H, *J* = 7.8), 7.92 (*d*, 1H, *J* = 7.8), 7.96 (*d*, 2H, *J* = 8.1), 8.20 (*s*, 1H), 8.29 (*d*, 1H, *J* = 8.3), 12.02 (*s*, 1H). Analysis calculated for C_21_H_22_N_2_O_2_S: C, 68.83; H, 6.05; N, 7.64. Found: C, 68.88; H, 5.99; N, 7.67.

**Spectroscopic and physical data for (II):** M.p. 478–479 K. FT–IR (ν_max_, cm^−1^): 3090, 1720, 1600, 1545, 1483, 1352, 1244, 1071. ^1^H NMR (600 MHz, DMSO-*d*_6_, 304 K): *δ* = 1.31 (*t*, 3H, *J* = 7.1), 4.23 (*q*, 2H, *J* = 7.1), 7.23 (*t*, 1H, *J* = 8.0), 7.49–7.64 (*m*, 1H), 7.83 (*t*, 1H, *J* = 8.0), 7.99 (*d*, 1H, *J* = 7.9), 8.28 (*d*, 1H, *J* = 7.5), 8.48 (*d*, 1H, *J* = 7.9), 8.85 (*s*, 1H), 11.65 (*s*, 1H). Analysis calculated for C_18_H_15_N_3_O_4_S: C, 58.53; H, 4.09; N, 11.38. Found: C, 58.59; H, 4.13; N, 11.47.

## Refinement   

Crystal data, data collection and structure refinement details are summarized in Table 3 ▸. X-ray diffraction studies were carried out on the ‘Belok’ beamline (λ = 0.96990 Å) of the National Research Center ‘Kurchatov Institute’ (Moscow, Russian Federation) using a MAR CCD detector. For each compound, a total of 360 images were collected using an oscillation range of 1.0° (φ scan mode) and corrected for absorption using the *SCAL*A program (Evans, 2006 ▸). The data were indexed, integrated and scaled using the utility *i*MOSFLM in the program *CCP4* (Battye *et al.*, 2011 ▸).

The hydrogen atoms of the amino groups were localized in difference-Fourier maps and refined in isotropic approximation with the constraint *U*_iso_(H) = 1.2*U*_eq_(N). The other hydrogen atoms were placed in calculated positions with C—H = 0.95–1.00 Å and refined using a riding model with *U*_iso_(H) = 1.5*U*_eq_(C) for the methyl group and 1.2*U*_eq_(C) for the other groups.

## Supplementary Material

Crystal structure: contains datablock(s) global, I, II. DOI: 10.1107/S2056989016013104/hb7607sup1.cif

Structure factors: contains datablock(s) I. DOI: 10.1107/S2056989016013104/hb7607Isup2.hkl

Structure factors: contains datablock(s) II. DOI: 10.1107/S2056989016013104/hb7607IIsup3.hkl

Supporting information file. DOI: 10.1107/S2056989016013104/hb7607Isup4.cml

Supporting information file. DOI: 10.1107/S2056989016013104/hb7607IIsup5.cml

CCDC references: 1499045, 1499044

Additional supporting information:  crystallographic information; 3D view; checkCIF report

## Figures and Tables

**Figure 2:**
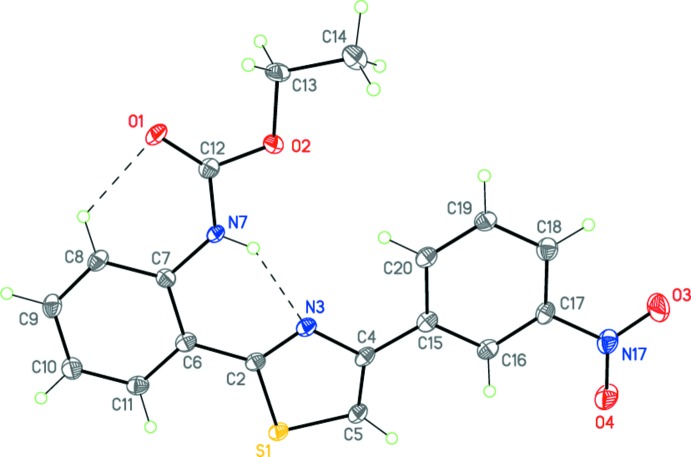
The mol­ecular structure of (II). Displacement ellipsoids are shown at the 50% probability level. Dashed lines indicate the intra­molecular hydrogen bonds. H atoms are presented as small spheres of arbitrary radius.

**Table 1:** Hydrogen-bond geometry (Å, °) for (I)

*D*—H⋯*A*	*D*—H	H⋯*A*	*D*⋯*A*	*D*—H⋯*A*
N7—H7⋯N3	0.97 (2)	1.84 (2)	2.682 (3)	144 (2)
C8—H8⋯O1	0.95	2.32	2.954 (3)	124

**Table 2:** Hydrogen-bond geometry (Å, °) for (II)

*D*—H⋯*A*	*D*—H	H⋯*A*	*D*⋯*A*	*D*—H⋯*A*
C5—H5⋯O1^i^	0.95	2.32	3.260 (3)	168
N7—H7⋯N3	0.80 (3)	1.98 (3)	2.672 (3)	144 (3)
C8—H8⋯O1	0.95	2.32	2.946 (3)	123

Symmetry code: (i) 
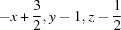
.

**Table 3:** Experimental details

	(I)	(II)
Crystal data		
Chemical formula	C_21_H_22_N_2_O_2_S	C_18_H_15_N_3_O_4_S
*M*_r_	366.47	369.39
Crystal system, space group	Orthorhombic, *P*2_1_2_1_2_1_	Orthorhombic, *P* *c* *a*2_1_
Temperature (K)	100	100
*a*, *b*, *c* (Å)	5.4534 (11), 17.203 (3), 20.060 (4)	23.840 (5), 9.7401 (19), 7.1403 (14)
*V* (Å^3^)	1881.9 (6)	1658.0 (6)
*Z*	4	4
Radiation type	Synchrotron, λ = 0.96990 Å	Synchrotron, λ = 0.96990 Å
μ (mm^−1^)	0.43	0.52
Crystal size (mm)	0.20 × 0.15 × 0.10	0.20 × 0.05 × 0.03

Data collection		
Diffractometer	MAR CCD	MAR CCD
Absorption correction	Multi-scan (*SCALA*; Evans, 2006 ▸)	Multi-scan (*SCALA*; Evans, 2006 ▸)
*T*_min_, *T*_max_	0.910, 0.950	0.890, 0.980
No. of measured, independent and observed [*I* > 2σ(*I*)] reflections	13728, 3971, 3052	13537, 3464, 3127
*R*_int_	0.095	0.073
(sin θ/λ)_max_ (Å^−1^)	0.641	0.641

Refinement		
*R*[*F* ^2^ > 2σ(*F* ^2^)], *wR*(*F* ^2^), *S*	0.052, 0.123, 0.96	0.037, 0.090, 1.05
No. of reflections	3971	3464
No. of parameters	242	240
No. of restraints	0	1
H-atom treatment	H atoms treated by a mixture of independent and constrained refinement	H atoms treated by a mixture of independent and constrained refinement
Δρ_max_, Δρ_min_ (e Å^−3^)	0.34, −0.39	0.25, −0.33
Absolute structure	Flack *x* determined using 997 quotients [(*I* ^+^)−(*I* ^−^)]/[(*I* ^+^)+(*I* ^−^)] (Parsons *et al.*, 2013 ▸)	Flack *x* determined using 1290 quotients [(*I* ^+^)−(*I* ^−^)]/[(*I* ^+^)+(*I* ^−^)] (Parsons *et al.*, 2013 ▸)
Absolute structure parameter	−0.01 (4)	0.39 (2)

Computer programs: *Automar* (MarXperts, 2015 ▸), *iMosflm* (Battye *et al.*, 2011 ▸), *SHELXS97* and *SHELXTL* (Sheldrick, 2008 ▸) and *SHELXL2014* (Sheldrick, 2015 ▸).

## supplementary crystallographic information

### (I) Ethyl {2-[4-(4-isopropylphenyl)thiazol-2-yl]phenyl}carbamate Crystal data

**Table:**

C_21_H_22_N_2_O_2_S	*D*_x_ = 1.293 Mg m^−^^3^
*M**_r_* = 366.47	Synchrotron radiation, λ = 0.96990 Å
Orthorhombic, *P*2_1_2_1_2_1_	Cell parameters from 500 reflections
*a* = 5.4534 (11) Å	θ = 3.5–35.0°
*b* = 17.203 (3) Å	µ = 0.43 mm^−^^1^
*c* = 20.060 (4) Å	*T* = 100 K
*V* = 1881.9 (6) Å^3^	Prism, colourless
*Z* = 4	0.20 × 0.15 × 0.10 mm
*F*(000) = 776	

### (I) Ethyl {2-[4-(4-isopropylphenyl)thiazol-2-yl]phenyl}carbamate Data collection

**Table:**

MAR CCD diffractometer	3052 reflections with *I* > 2σ(*I*)
phi scan	*R*_int_ = 0.095
Absorption correction: multi-scan (SCALA; Evans, 2006)	θ_max_ = 38.5°, θ_min_ = 3.5°
*T*_min_ = 0.910, *T*_max_ = 0.950	*h* = −6→6
13728 measured reflections	*k* = −20→21
3971 independent reflections	*l* = −25→25

### (I) Ethyl {2-[4-(4-isopropylphenyl)thiazol-2-yl]phenyl}carbamate Refinement

**Table:**

Refinement on *F*^2^	Hydrogen site location: mixed
Least-squares matrix: full	H atoms treated by a mixture of independent and constrained refinement
*R*[*F*^2^ > 2σ(*F*^2^)] = 0.052	*w* = 1/[σ^2^(*F*_o_^2^) + (0.004*P*)^2^] where *P* = (*F*_o_^2^ + 2*F*_c_^2^)/3
*wR*(*F*^2^) = 0.123	(Δ/σ)_max_ < 0.001
*S* = 0.96	Δρ_max_ = 0.34 e Å^−^^3^
3971 reflections	Δρ_min_ = −0.39 e Å^−^^3^
242 parameters	Extinction correction: SHELXL2014 (Sheldrick, 2015), Fc^*^=kFc[1+0.001xFc^2^λ^3^/sin(2θ)]^-1/4^
0 restraints	Extinction coefficient: 0.0210 (11)
Primary atom site location: difference Fourier map	Absolute structure: Flack *x* determined using 997 quotients [(*I*^+^)-(*I*^-^)]/[(*I*^+^)+(*I*^-^)] (Parsons *et al.*, 2013)
Secondary atom site location: difference Fourier map	Absolute structure parameter: −0.01 (4)

### (I) Ethyl {2-[4-(4-isopropylphenyl)thiazol-2-yl]phenyl}carbamate Special details

**Table:**

Geometry. All esds (except the esd in the dihedral angle between two l.s. planes) are estimated using the full covariance matrix. The cell esds are taken into account individually in the estimation of esds in distances, angles and torsion angles; correlations between esds in cell parameters are only used when they are defined by crystal symmetry. An approximate (isotropic) treatment of cell esds is used for estimating esds involving l.s. planes.

### (I) Ethyl {2-[4-(4-isopropylphenyl)thiazol-2-yl]phenyl}carbamate Fractional atomic coordinates and isotropic or equivalent isotropic displacement parameters (Å^2^)

**Table:**

	*x*	*y*	*z*	*U*_iso_*/*U*_eq_	
S1	0.24911 (12)	0.47012 (3)	0.42460 (3)	0.03010 (16)	
O1	1.2359 (3)	0.58830 (10)	0.61640 (9)	0.0352 (5)	
O2	1.0633 (3)	0.47367 (9)	0.64975 (8)	0.0288 (4)	
C2	0.4926 (5)	0.49392 (14)	0.47800 (12)	0.0259 (6)	
N3	0.5248 (4)	0.44158 (11)	0.52534 (10)	0.0257 (5)	
C4	0.3588 (4)	0.38088 (13)	0.52171 (12)	0.0239 (6)	
C5	0.1972 (5)	0.38674 (14)	0.47016 (13)	0.0287 (7)	
H5	0.0725	0.3498	0.4606	0.034*	
C6	0.6380 (5)	0.56568 (14)	0.46990 (13)	0.0268 (6)	
C7	0.8381 (5)	0.58484 (14)	0.51199 (12)	0.0257 (6)	
N7	0.8934 (4)	0.53396 (11)	0.56480 (10)	0.0280 (5)	
H7	0.787 (4)	0.4888 (14)	0.5664 (12)	0.034*	
C8	0.9726 (5)	0.65255 (14)	0.50006 (13)	0.0319 (7)	
H8	1.1061	0.6655	0.5284	0.038*	
C9	0.9130 (5)	0.70106 (15)	0.44715 (13)	0.0350 (7)	
H9	1.0076	0.7465	0.4392	0.042*	
C10	0.7167 (5)	0.68374 (15)	0.40576 (14)	0.0367 (7)	
H10	0.6744	0.7174	0.3700	0.044*	
C11	0.5833 (5)	0.61661 (14)	0.41741 (13)	0.0329 (7)	
H11	0.4498	0.6046	0.3888	0.039*	
C12	1.0813 (5)	0.53798 (14)	0.61028 (12)	0.0261 (6)	
C13	1.2512 (5)	0.46609 (14)	0.70045 (13)	0.0332 (7)	
H13A	1.2590	0.5138	0.7280	0.040*	
H13B	1.4134	0.4577	0.6795	0.040*	
C14	1.1827 (5)	0.39723 (15)	0.74298 (13)	0.0364 (8)	
H14A	1.1701	0.3508	0.7149	0.055*	
H14B	1.0247	0.4071	0.7646	0.055*	
H14C	1.3090	0.3891	0.7770	0.055*	
C15	0.3725 (4)	0.31803 (13)	0.57234 (12)	0.0230 (6)	
C16	0.5701 (5)	0.31297 (14)	0.61706 (12)	0.0262 (6)	
H16	0.7017	0.3489	0.6138	0.031*	
C17	0.5750 (5)	0.25610 (13)	0.66585 (13)	0.0265 (6)	
H17	0.7105	0.2544	0.6956	0.032*	
C18	0.3887 (5)	0.20128 (14)	0.67307 (12)	0.0257 (6)	
C19	0.1953 (5)	0.20502 (14)	0.62699 (13)	0.0279 (7)	
H19	0.0673	0.1676	0.6293	0.033*	
C20	0.1868 (4)	0.26245 (13)	0.57785 (13)	0.0249 (6)	
H20	0.0526	0.2638	0.5477	0.030*	
C21	0.3936 (5)	0.14340 (14)	0.73046 (14)	0.0323 (7)	
H21	0.5692	0.1301	0.7391	0.039*	
C22	0.2582 (6)	0.06725 (13)	0.71601 (15)	0.0391 (7)	
H22A	0.0830	0.0779	0.7101	0.059*	
H22B	0.2810	0.0313	0.7534	0.059*	
H22C	0.3241	0.0438	0.6752	0.059*	
C23	0.2919 (6)	0.18119 (16)	0.79435 (14)	0.0446 (9)	
H23A	0.3763	0.2306	0.8024	0.067*	
H23B	0.3188	0.1463	0.8323	0.067*	
H23C	0.1157	0.1907	0.7891	0.067*	

### (I) Ethyl {2-[4-(4-isopropylphenyl)thiazol-2-yl]phenyl}carbamate Atomic displacement parameters (Å^2^)

**Table:**

	*U*^11^	*U*^22^	*U*^33^	*U*^12^	*U*^13^	*U*^23^
S1	0.0324 (3)	0.0306 (3)	0.0273 (3)	0.0019 (3)	−0.0031 (3)	−0.0005 (3)
O1	0.0338 (10)	0.0280 (8)	0.0438 (10)	−0.0087 (9)	−0.0062 (9)	0.0005 (8)
O2	0.0354 (10)	0.0240 (8)	0.0269 (9)	−0.0026 (8)	−0.0049 (8)	0.0034 (8)
C2	0.0264 (13)	0.0239 (12)	0.0275 (13)	0.0034 (12)	0.0057 (11)	−0.0012 (10)
N3	0.0304 (12)	0.0215 (9)	0.0252 (11)	−0.0013 (9)	0.0028 (9)	−0.0015 (8)
C4	0.0239 (13)	0.0204 (11)	0.0274 (13)	−0.0015 (11)	0.0020 (11)	−0.0061 (10)
C5	0.0271 (15)	0.0297 (12)	0.0293 (13)	0.0018 (12)	−0.0014 (11)	−0.0068 (11)
C6	0.0330 (15)	0.0250 (12)	0.0224 (13)	0.0036 (11)	0.0037 (11)	0.0001 (10)
C7	0.0312 (14)	0.0207 (11)	0.0253 (13)	0.0032 (11)	0.0040 (11)	0.0013 (10)
N7	0.0304 (12)	0.0245 (10)	0.0292 (11)	−0.0036 (10)	−0.0060 (10)	0.0024 (9)
C8	0.0353 (16)	0.0238 (12)	0.0367 (15)	−0.0024 (12)	0.0019 (13)	−0.0004 (11)
C9	0.0432 (16)	0.0241 (12)	0.0378 (15)	−0.0040 (13)	0.0107 (14)	0.0012 (12)
C10	0.0457 (17)	0.0308 (13)	0.0336 (14)	0.0004 (14)	0.0030 (14)	0.0080 (11)
C11	0.0376 (15)	0.0328 (13)	0.0282 (14)	0.0020 (13)	−0.0008 (13)	0.0019 (12)
C12	0.0275 (14)	0.0236 (12)	0.0274 (13)	0.0015 (12)	0.0010 (11)	−0.0013 (10)
C13	0.0336 (15)	0.0329 (13)	0.0330 (13)	0.0064 (15)	−0.0064 (13)	−0.0056 (11)
C14	0.0428 (18)	0.0380 (14)	0.0284 (14)	0.0119 (13)	−0.0025 (12)	−0.0002 (12)
C15	0.0221 (13)	0.0235 (11)	0.0234 (12)	0.0025 (10)	0.0014 (11)	−0.0056 (10)
C16	0.0252 (14)	0.0251 (12)	0.0283 (13)	−0.0009 (12)	0.0007 (11)	−0.0033 (10)
C17	0.0238 (13)	0.0261 (12)	0.0296 (13)	0.0017 (12)	0.0002 (12)	−0.0023 (11)
C18	0.0222 (13)	0.0224 (11)	0.0325 (14)	0.0026 (12)	0.0028 (12)	−0.0033 (11)
C19	0.0252 (15)	0.0227 (11)	0.0357 (14)	−0.0007 (12)	0.0013 (11)	−0.0035 (11)
C20	0.0233 (13)	0.0248 (11)	0.0267 (12)	0.0007 (10)	0.0006 (11)	−0.0056 (10)
C21	0.0278 (14)	0.0268 (12)	0.0424 (16)	−0.0013 (12)	−0.0010 (13)	0.0044 (12)
C22	0.0382 (16)	0.0303 (13)	0.0489 (16)	−0.0025 (14)	−0.0002 (16)	0.0083 (12)
C23	0.059 (2)	0.0388 (14)	0.0361 (16)	−0.0010 (16)	−0.0014 (15)	0.0080 (12)

### (I) Ethyl {2-[4-(4-isopropylphenyl)thiazol-2-yl]phenyl}carbamate Geometric parameters (Å, º)

**Table:**

S1—C5	1.724 (3)	C13—H13A	0.9900
S1—C2	1.755 (3)	C13—H13B	0.9900
O1—C12	1.215 (3)	C14—H14A	0.9800
O2—C12	1.364 (3)	C14—H14B	0.9800
O2—C13	1.449 (3)	C14—H14C	0.9800
C2—N3	1.320 (3)	C15—C20	1.397 (3)
C2—C6	1.476 (3)	C15—C16	1.405 (3)
N3—C4	1.384 (3)	C16—C17	1.384 (3)
C4—C5	1.362 (3)	C16—H16	0.9500
C4—C15	1.485 (3)	C17—C18	1.394 (3)
C5—H5	0.9500	C17—H17	0.9500
C6—C11	1.402 (3)	C18—C19	1.404 (4)
C6—C7	1.419 (4)	C18—C21	1.522 (4)
C7—C8	1.397 (3)	C19—C20	1.396 (3)
C7—N7	1.407 (3)	C19—H19	0.9500
N7—C12	1.374 (3)	C20—H20	0.9500
N7—H7	0.97 (2)	C21—C22	1.531 (4)
C8—C9	1.389 (4)	C21—C23	1.540 (4)
C8—H8	0.9500	C21—H21	1.0000
C9—C10	1.387 (4)	C22—H22A	0.9800
C9—H9	0.9500	C22—H22B	0.9800
C10—C11	1.385 (4)	C22—H22C	0.9800
C10—H10	0.9500	C23—H23A	0.9800
C11—H11	0.9500	C23—H23B	0.9800
C13—C14	1.507 (4)	C23—H23C	0.9800
			
C5—S1—C2	89.70 (12)	C13—C14—H14A	109.5
C12—O2—C13	115.43 (18)	C13—C14—H14B	109.5
N3—C2—C6	125.3 (2)	H14A—C14—H14B	109.5
N3—C2—S1	112.38 (18)	C13—C14—H14C	109.5
C6—C2—S1	122.31 (19)	H14A—C14—H14C	109.5
C2—N3—C4	112.9 (2)	H14B—C14—H14C	109.5
C5—C4—N3	114.0 (2)	C20—C15—C16	117.6 (2)
C5—C4—C15	127.3 (2)	C20—C15—C4	121.0 (2)
N3—C4—C15	118.7 (2)	C16—C15—C4	121.4 (2)
C4—C5—S1	110.96 (19)	C17—C16—C15	120.7 (2)
C4—C5—H5	124.5	C17—C16—H16	119.7
S1—C5—H5	124.5	C15—C16—H16	119.7
C11—C6—C7	117.7 (2)	C16—C17—C18	122.5 (2)
C11—C6—C2	119.4 (2)	C16—C17—H17	118.7
C7—C6—C2	122.8 (2)	C18—C17—H17	118.7
C8—C7—N7	122.4 (2)	C17—C18—C19	116.6 (2)
C8—C7—C6	119.7 (2)	C17—C18—C21	120.6 (2)
N7—C7—C6	117.9 (2)	C19—C18—C21	122.8 (2)
C12—N7—C7	128.9 (2)	C20—C19—C18	121.5 (2)
C12—N7—H7	117.8 (15)	C20—C19—H19	119.3
C7—N7—H7	113.2 (15)	C18—C19—H19	119.3
C9—C8—C7	120.6 (3)	C19—C20—C15	121.1 (2)
C9—C8—H8	119.7	C19—C20—H20	119.5
C7—C8—H8	119.7	C15—C20—H20	119.5
C10—C9—C8	120.6 (2)	C18—C21—C22	114.1 (2)
C10—C9—H9	119.7	C18—C21—C23	110.3 (2)
C8—C9—H9	119.7	C22—C21—C23	110.2 (2)
C11—C10—C9	118.9 (3)	C18—C21—H21	107.3
C11—C10—H10	120.5	C22—C21—H21	107.3
C9—C10—H10	120.5	C23—C21—H21	107.3
C10—C11—C6	122.4 (3)	C21—C22—H22A	109.5
C10—C11—H11	118.8	C21—C22—H22B	109.5
C6—C11—H11	118.8	H22A—C22—H22B	109.5
O1—C12—O2	124.7 (2)	C21—C22—H22C	109.5
O1—C12—N7	128.4 (2)	H22A—C22—H22C	109.5
O2—C12—N7	106.9 (2)	H22B—C22—H22C	109.5
O2—C13—C14	107.0 (2)	C21—C23—H23A	109.5
O2—C13—H13A	110.3	C21—C23—H23B	109.5
C14—C13—H13A	110.3	H23A—C23—H23B	109.5
O2—C13—H13B	110.3	C21—C23—H23C	109.5
C14—C13—H13B	110.3	H23A—C23—H23C	109.5
H13A—C13—H13B	108.6	H23B—C23—H23C	109.5
			
C5—S1—C2—N3	0.05 (19)	C2—C6—C11—C10	−178.1 (2)
C5—S1—C2—C6	−179.0 (2)	C13—O2—C12—O1	−2.2 (3)
C6—C2—N3—C4	178.7 (2)	C13—O2—C12—N7	178.51 (19)
S1—C2—N3—C4	−0.3 (3)	C7—N7—C12—O1	3.2 (4)
C2—N3—C4—C5	0.4 (3)	C7—N7—C12—O2	−177.5 (2)
C2—N3—C4—C15	−179.5 (2)	C12—O2—C13—C14	173.7 (2)
N3—C4—C5—S1	−0.4 (3)	C5—C4—C15—C20	−10.5 (4)
C15—C4—C5—S1	179.5 (2)	N3—C4—C15—C20	169.4 (2)
C2—S1—C5—C4	0.19 (19)	C5—C4—C15—C16	170.5 (2)
N3—C2—C6—C11	−179.7 (2)	N3—C4—C15—C16	−9.5 (3)
S1—C2—C6—C11	−0.8 (3)	C20—C15—C16—C17	−1.8 (3)
N3—C2—C6—C7	2.3 (4)	C4—C15—C16—C17	177.1 (2)
S1—C2—C6—C7	−178.8 (2)	C15—C16—C17—C18	0.4 (4)
C11—C6—C7—C8	−0.1 (4)	C16—C17—C18—C19	1.5 (4)
C2—C6—C7—C8	177.9 (2)	C16—C17—C18—C21	−175.7 (2)
C11—C6—C7—N7	179.9 (2)	C17—C18—C19—C20	−2.0 (4)
C2—C6—C7—N7	−2.1 (4)	C21—C18—C19—C20	175.1 (2)
C8—C7—N7—C12	−2.8 (4)	C18—C19—C20—C15	0.7 (4)
C6—C7—N7—C12	177.2 (2)	C16—C15—C20—C19	1.3 (3)
N7—C7—C8—C9	179.6 (2)	C4—C15—C20—C19	−177.7 (2)
C6—C7—C8—C9	−0.4 (4)	C17—C18—C21—C22	−153.4 (2)
C7—C8—C9—C10	0.9 (4)	C19—C18—C21—C22	29.5 (3)
C8—C9—C10—C11	−1.0 (4)	C17—C18—C21—C23	82.0 (3)
C9—C10—C11—C6	0.5 (4)	C19—C18—C21—C23	−95.1 (3)
C7—C6—C11—C10	0.0 (4)		

### (I) Ethyl {2-[4-(4-isopropylphenyl)thiazol-2-yl]phenyl}carbamate Hydrogen-bond geometry (Å, º)

**Table:**

*D*—H···*A*	*D*—H	H···*A*	*D*···*A*	*D*—H···*A*
N7—H7···N3	0.97 (2)	1.84 (2)	2.682 (3)	144 (2)
C8—H8···O1	0.95	2.32	2.954 (3)	124

### (II) Ethyl {2-[4-(3-nitrophenyl)thiazol-2-yl]phenyl}carbamate Crystal data

**Table:**

C_18_H_15_N_3_O_4_S	*D*_x_ = 1.480 Mg m^−^^3^
*M**_r_* = 369.39	Synchrotron radiation, λ = 0.96990 Å
Orthorhombic, *P**c**a*2_1_	Cell parameters from 600 reflections
*a* = 23.840 (5) Å	θ = 3.7–37.0°
*b* = 9.7401 (19) Å	µ = 0.52 mm^−^^1^
*c* = 7.1403 (14) Å	*T* = 100 K
*V* = 1658.0 (6) Å^3^	Needle, colourless
*Z* = 4	0.20 × 0.05 × 0.03 mm
*F*(000) = 768	

### (II) Ethyl {2-[4-(3-nitrophenyl)thiazol-2-yl]phenyl}carbamate Data collection

**Table:**

MAR CCD diffractometer	3127 reflections with *I* > 2σ(*I*)
phi scan	*R*_int_ = 0.073
Absorption correction: multi-scan (SCALA; Evans, 2006)	θ_max_ = 38.5°, θ_min_ = 3.7°
*T*_min_ = 0.890, *T*_max_ = 0.980	*h* = −29→29
13537 measured reflections	*k* = −12→12
3464 independent reflections	*l* = −9→8

### (II) Ethyl {2-[4-(3-nitrophenyl)thiazol-2-yl]phenyl}carbamate Refinement

**Table:**

Refinement on *F*^2^	Hydrogen site location: mixed
Least-squares matrix: full	H atoms treated by a mixture of independent and constrained refinement
*R*[*F*^2^ > 2σ(*F*^2^)] = 0.037	*w* = 1/[σ^2^(*F*_o_^2^) + (0.0408*P*)^2^] where *P* = (*F*_o_^2^ + 2*F*_c_^2^)/3
*wR*(*F*^2^) = 0.090	(Δ/σ)_max_ < 0.001
*S* = 1.05	Δρ_max_ = 0.25 e Å^−^^3^
3464 reflections	Δρ_min_ = −0.33 e Å^−^^3^
240 parameters	Extinction correction: SHELXL2014 (Sheldrick, 2015), Fc^*^=kFc[1+0.001xFc^2^λ^3^/sin(2θ)]^-1/4^
1 restraint	Extinction coefficient: 0.010 (2)
Primary atom site location: difference Fourier map	Absolute structure: Flack *x* determined using 1290 quotients [(*I*^+^)-(*I*^-^)]/[(*I*^+^)+(*I*^-^)] (Parsons *et al.*, 2013)
Secondary atom site location: difference Fourier map	Absolute structure parameter: 0.39 (2)

### (II) Ethyl {2-[4-(3-nitrophenyl)thiazol-2-yl]phenyl}carbamate Special details

**Table:**

Geometry. All esds (except the esd in the dihedral angle between two l.s. planes) are estimated using the full covariance matrix. The cell esds are taken into account individually in the estimation of esds in distances, angles and torsion angles; correlations between esds in cell parameters are only used when they are defined by crystal symmetry. An approximate (isotropic) treatment of cell esds is used for estimating esds involving l.s. planes.

### (II) Ethyl {2-[4-(3-nitrophenyl)thiazol-2-yl]phenyl}carbamate Fractional atomic coordinates and isotropic or equivalent isotropic displacement parameters (Å^2^)

**Table:**

	*x*	*y*	*z*	*U*_iso_*/*U*_eq_	
S1	0.82320 (2)	0.08743 (5)	0.40138 (11)	0.01687 (18)	
O1	0.78056 (7)	0.74689 (17)	0.7251 (3)	0.0221 (5)	
O2	0.69969 (7)	0.63003 (18)	0.6617 (3)	0.0183 (4)	
O3	0.47989 (8)	−0.0296 (2)	0.3607 (4)	0.0367 (6)	
O4	0.55366 (8)	−0.14517 (18)	0.2763 (3)	0.0279 (5)	
C2	0.80673 (11)	0.2564 (2)	0.4636 (4)	0.0148 (5)	
N3	0.75204 (10)	0.27926 (18)	0.4676 (3)	0.0155 (5)	
C4	0.72122 (10)	0.1639 (2)	0.4202 (4)	0.0152 (5)	
C5	0.75302 (11)	0.0509 (2)	0.3800 (4)	0.0172 (5)	
H5	0.7383	−0.0360	0.3449	0.021*	
C6	0.85019 (10)	0.3582 (2)	0.5131 (4)	0.0160 (5)	
C7	0.83664 (11)	0.4896 (2)	0.5898 (4)	0.0148 (6)	
N7	0.77977 (9)	0.5244 (2)	0.6077 (3)	0.0163 (5)	
H7	0.7579 (13)	0.467 (3)	0.575 (5)	0.020*	
C8	0.88015 (12)	0.5776 (2)	0.6456 (4)	0.0180 (6)	
H8	0.8715	0.6643	0.6992	0.022*	
C9	0.93587 (11)	0.5388 (3)	0.6230 (4)	0.0192 (6)	
H9	0.9649	0.5992	0.6622	0.023*	
C10	0.94981 (12)	0.4124 (2)	0.5438 (4)	0.0196 (6)	
H10	0.9880	0.3873	0.5269	0.024*	
C11	0.90696 (11)	0.3236 (2)	0.4899 (4)	0.0179 (6)	
H11	0.9164	0.2375	0.4360	0.021*	
C12	0.75647 (11)	0.6442 (2)	0.6700 (4)	0.0155 (5)	
C13	0.66740 (11)	0.7511 (3)	0.7127 (4)	0.0202 (6)	
H13A	0.6795	0.8311	0.6371	0.024*	
H13B	0.6729	0.7731	0.8468	0.024*	
C14	0.60652 (11)	0.7183 (3)	0.6745 (4)	0.0240 (6)	
H14A	0.6015	0.6987	0.5409	0.036*	
H14B	0.5832	0.7971	0.7096	0.036*	
H14C	0.5953	0.6380	0.7482	0.036*	
C15	0.65919 (10)	0.1740 (2)	0.4257 (4)	0.0157 (5)	
C16	0.62493 (11)	0.0627 (2)	0.3740 (4)	0.0168 (6)	
H16	0.6410	−0.0208	0.3311	0.020*	
C17	0.56702 (10)	0.0777 (2)	0.3873 (4)	0.0173 (6)	
N17	0.53118 (10)	−0.0404 (2)	0.3375 (4)	0.0216 (5)	
C18	0.54099 (11)	0.1977 (3)	0.4468 (4)	0.0198 (6)	
H18	0.5013	0.2042	0.4547	0.024*	
C19	0.57550 (11)	0.3088 (2)	0.4949 (4)	0.0188 (6)	
H19	0.5591	0.3927	0.5351	0.023*	
C20	0.63338 (11)	0.2970 (2)	0.4843 (4)	0.0179 (6)	
H20	0.6561	0.3734	0.5173	0.021*	

### (II) Ethyl {2-[4-(3-nitrophenyl)thiazol-2-yl]phenyl}carbamate Atomic displacement parameters (Å^2^)

**Table:**

	*U*^11^	*U*^22^	*U*^33^	*U*^12^	*U*^13^	*U*^23^
S1	0.0182 (3)	0.0103 (3)	0.0221 (4)	0.0010 (2)	−0.0001 (3)	−0.0034 (3)
O1	0.0226 (10)	0.0118 (9)	0.0320 (13)	−0.0024 (7)	−0.0008 (8)	−0.0069 (8)
O2	0.0139 (10)	0.0151 (9)	0.0260 (11)	0.0009 (7)	0.0029 (8)	−0.0035 (8)
O3	0.0206 (11)	0.0298 (11)	0.0596 (19)	−0.0090 (8)	0.0045 (11)	−0.0107 (11)
O4	0.0282 (11)	0.0123 (9)	0.0432 (14)	0.0024 (8)	−0.0086 (10)	−0.0063 (9)
C2	0.0186 (13)	0.0127 (11)	0.0131 (14)	0.0002 (10)	−0.0006 (9)	0.0000 (10)
N3	0.0187 (11)	0.0117 (9)	0.0160 (13)	−0.0007 (8)	0.0000 (9)	−0.0010 (8)
C4	0.0213 (12)	0.0104 (11)	0.0138 (14)	−0.0023 (9)	−0.0019 (11)	0.0003 (10)
C5	0.0206 (12)	0.0128 (10)	0.0181 (15)	−0.0025 (10)	−0.0009 (12)	−0.0018 (11)
C6	0.0199 (14)	0.0119 (12)	0.0161 (15)	0.0011 (10)	−0.0007 (11)	0.0014 (10)
C7	0.0152 (12)	0.0128 (12)	0.0165 (16)	0.0012 (9)	0.0014 (10)	0.0017 (10)
N7	0.0148 (11)	0.0094 (10)	0.0248 (14)	−0.0014 (8)	0.0019 (9)	−0.0050 (9)
C8	0.0219 (14)	0.0105 (12)	0.0215 (16)	−0.0008 (9)	−0.0019 (11)	−0.0002 (11)
C9	0.0185 (13)	0.0152 (12)	0.0240 (16)	−0.0027 (10)	−0.0013 (11)	0.0032 (11)
C10	0.0182 (14)	0.0176 (14)	0.0231 (16)	0.0003 (9)	0.0001 (11)	0.0009 (11)
C11	0.0210 (13)	0.0133 (12)	0.0194 (15)	0.0027 (9)	0.0010 (11)	−0.0001 (10)
C12	0.0184 (13)	0.0104 (11)	0.0177 (14)	0.0003 (10)	0.0004 (11)	0.0026 (10)
C13	0.0188 (13)	0.0136 (13)	0.0282 (17)	0.0063 (10)	0.0021 (12)	−0.0010 (12)
C14	0.0187 (13)	0.0273 (15)	0.0259 (18)	0.0029 (11)	0.0015 (11)	−0.0001 (12)
C15	0.0207 (13)	0.0110 (11)	0.0153 (14)	−0.0008 (9)	−0.0010 (11)	0.0029 (10)
C16	0.0212 (13)	0.0121 (11)	0.0170 (16)	0.0004 (9)	−0.0013 (11)	0.0008 (10)
C17	0.0200 (12)	0.0120 (11)	0.0200 (16)	−0.0050 (9)	−0.0009 (13)	0.0011 (11)
N17	0.0222 (12)	0.0145 (10)	0.0282 (15)	−0.0028 (9)	−0.0037 (10)	0.0005 (10)
C18	0.0199 (13)	0.0179 (13)	0.0215 (17)	−0.0013 (10)	−0.0011 (11)	0.0010 (11)
C19	0.0208 (13)	0.0115 (12)	0.0243 (15)	0.0006 (10)	0.0012 (11)	−0.0019 (11)
C20	0.0211 (13)	0.0133 (12)	0.0192 (14)	−0.0010 (10)	−0.0006 (11)	−0.0009 (10)

### (II) Ethyl {2-[4-(3-nitrophenyl)thiazol-2-yl]phenyl}carbamate Geometric parameters (Å, º)

**Table:**

S1—C5	1.717 (3)	C9—H9	0.9500
S1—C2	1.750 (2)	C10—C11	1.393 (4)
O1—C12	1.218 (3)	C10—H10	0.9500
O2—C12	1.362 (3)	C11—H11	0.9500
O2—C13	1.455 (3)	C13—C14	1.511 (4)
O3—N17	1.239 (3)	C13—H13A	0.9900
O4—N17	1.233 (3)	C13—H13B	0.9900
C2—N3	1.323 (3)	C14—H14A	0.9800
C2—C6	1.477 (3)	C14—H14B	0.9800
N3—C4	1.385 (3)	C14—H14C	0.9800
C4—C5	1.367 (3)	C15—C16	1.406 (3)
C4—C15	1.482 (3)	C15—C20	1.411 (3)
C5—H5	0.9500	C16—C17	1.391 (3)
C6—C11	1.405 (3)	C16—H16	0.9500
C6—C7	1.429 (3)	C17—C18	1.390 (3)
C7—N7	1.404 (3)	C17—N17	1.477 (3)
C7—C8	1.403 (4)	C18—C19	1.402 (4)
N7—C12	1.367 (3)	C18—H18	0.9500
N7—H7	0.80 (3)	C19—C20	1.387 (4)
C8—C9	1.391 (4)	C19—H19	0.9500
C8—H8	0.9500	C20—H20	0.9500
C9—C10	1.395 (4)		
			
C5—S1—C2	89.94 (12)	O2—C12—N7	107.6 (2)
C12—O2—C13	115.65 (18)	O2—C13—C14	107.0 (2)
N3—C2—C6	125.0 (2)	O2—C13—H13A	110.3
N3—C2—S1	112.64 (17)	C14—C13—H13A	110.3
C6—C2—S1	122.34 (19)	O2—C13—H13B	110.3
C2—N3—C4	112.41 (19)	C14—C13—H13B	110.3
C5—C4—N3	114.2 (2)	H13A—C13—H13B	108.6
C5—C4—C15	127.8 (2)	C13—C14—H14A	109.5
N3—C4—C15	118.0 (2)	C13—C14—H14B	109.5
C4—C5—S1	110.78 (18)	H14A—C14—H14B	109.5
C4—C5—H5	124.6	C13—C14—H14C	109.5
S1—C5—H5	124.6	H14A—C14—H14C	109.5
C11—C6—C7	118.6 (2)	H14B—C14—H14C	109.5
C11—C6—C2	119.1 (2)	C16—C15—C20	118.6 (2)
C7—C6—C2	122.3 (2)	C16—C15—C4	121.4 (2)
N7—C7—C8	122.7 (2)	C20—C15—C4	120.0 (2)
N7—C7—C6	118.0 (2)	C17—C16—C15	118.5 (2)
C8—C7—C6	119.3 (2)	C17—C16—H16	120.7
C12—N7—C7	128.9 (2)	C15—C16—H16	120.7
C12—N7—H7	116 (2)	C18—C17—C16	123.5 (2)
C7—N7—H7	116 (2)	C18—C17—N17	118.1 (2)
C9—C8—C7	120.5 (2)	C16—C17—N17	118.4 (2)
C9—C8—H8	119.8	O4—N17—O3	123.2 (2)
C7—C8—H8	119.8	O4—N17—C17	118.6 (2)
C8—C9—C10	121.0 (2)	O3—N17—C17	118.2 (2)
C8—C9—H9	119.5	C17—C18—C19	117.5 (2)
C10—C9—H9	119.5	C17—C18—H18	121.3
C11—C10—C9	119.0 (2)	C19—C18—H18	121.3
C11—C10—H10	120.5	C20—C19—C18	120.5 (2)
C9—C10—H10	120.5	C20—C19—H19	119.8
C10—C11—C6	121.7 (2)	C18—C19—H19	119.8
C10—C11—H11	119.2	C19—C20—C15	121.4 (2)
C6—C11—H11	119.2	C19—C20—H20	119.3
O1—C12—O2	124.5 (2)	C15—C20—H20	119.3
O1—C12—N7	127.9 (2)		
			
C5—S1—C2—N3	0.4 (2)	C2—C6—C11—C10	−176.4 (3)
C5—S1—C2—C6	178.0 (2)	C13—O2—C12—O1	3.8 (4)
C6—C2—N3—C4	−178.0 (2)	C13—O2—C12—N7	−176.5 (2)
S1—C2—N3—C4	−0.4 (3)	C7—N7—C12—O1	−0.6 (5)
C2—N3—C4—C5	0.2 (3)	C7—N7—C12—O2	179.8 (2)
C2—N3—C4—C15	177.9 (2)	C12—O2—C13—C14	174.2 (2)
N3—C4—C5—S1	0.1 (3)	C5—C4—C15—C16	−4.7 (5)
C15—C4—C5—S1	−177.3 (2)	N3—C4—C15—C16	178.0 (3)
C2—S1—C5—C4	−0.3 (2)	C5—C4—C15—C20	175.1 (3)
N3—C2—C6—C11	−175.5 (2)	N3—C4—C15—C20	−2.2 (4)
S1—C2—C6—C11	7.2 (4)	C20—C15—C16—C17	−1.5 (4)
N3—C2—C6—C7	6.7 (4)	C4—C15—C16—C17	178.3 (3)
S1—C2—C6—C7	−170.7 (2)	C15—C16—C17—C18	0.8 (4)
C11—C6—C7—N7	178.4 (2)	C15—C16—C17—N17	−178.5 (3)
C2—C6—C7—N7	−3.7 (4)	C18—C17—N17—O4	176.7 (3)
C11—C6—C7—C8	−2.3 (4)	C16—C17—N17—O4	−3.9 (4)
C2—C6—C7—C8	175.6 (2)	C18—C17—N17—O3	−3.9 (4)
C8—C7—N7—C12	3.2 (5)	C16—C17—N17—O3	175.5 (3)
C6—C7—N7—C12	−177.5 (3)	C16—C17—C18—C19	0.3 (4)
N7—C7—C8—C9	−179.3 (3)	N17—C17—C18—C19	179.6 (2)
C6—C7—C8—C9	1.3 (4)	C17—C18—C19—C20	−0.6 (4)
C7—C8—C9—C10	0.4 (4)	C18—C19—C20—C15	−0.1 (4)
C8—C9—C10—C11	−1.1 (4)	C16—C15—C20—C19	1.2 (4)
C9—C10—C11—C6	0.1 (4)	C4—C15—C20—C19	−178.6 (2)
C7—C6—C11—C10	1.6 (4)		

### (II) Ethyl {2-[4-(3-nitrophenyl)thiazol-2-yl]phenyl}carbamate Hydrogen-bond geometry (Å, º)

**Table:**

*D*—H···*A*	*D*—H	H···*A*	*D*···*A*	*D*—H···*A*
C5—H5···O1^i^	0.95	2.32	3.260 (3)	168
N7—H7···N3	0.80 (3)	1.98 (3)	2.672 (3)	144 (3)
C8—H8···O1	0.95	2.32	2.946 (3)	123

Symmetry code: (i) −*x*+3/2, *y*−1, *z*−1/2.

## References

[bb1] AaminaNaaz, Y., Sathiyaraj, S., Kalaimani, S., Nasar, A. S. & SubbiahPandi, A. (2015). *Acta Cryst.* E**71**, o969–o970. 10.1107/S2056989015021544 PMC4719927 26870555

[bb2] Battye, T. G. G., Kontogiannis, L., Johnson, O., Powell, H. R. & Leslie, A. G. W. (2011). *Acta Cryst.* D**67**, 271–281. 10.1107/S0907444910048675 PMC3069742 21460445

[bb3] Bérdy, J. (2005). *J. Antibiot.* **58**, 1–26. 10.1038/ja.2005.1 15813176

[bb4] Bister, B., Bischoff, D., Ströbele, M., Riedlinger, J., Reicke, A., Wolter, F., Bull, A. T., Zähner, H., Fiedler, H. P. & Süssmuth, R. D. (2004). *Angew. Chem. Int. Ed.* **43**, 2574–2576. 10.1002/anie.200353160 15127456

[bb5] Bull, A. T. & Stach, J. E. (2007). *Trends Microbiol.* **15**, 491–499. 10.1016/j.tim.2007.10.004 17997312

[bb6] Bunev, A. S., Rudakova, Y. I., Statsyuk, V. E., Ostapenko, G. I. & Khrustalev, V. N. (2014). *Acta Cryst.* E**70**, o139. 10.1107/S160053681400066X PMC3998304 24764865

[bb7] Carroll, A. R., Coll, J. C., Bourne, D. L., MacLeod, J. K., Ireland, C. M. & Bowden, B. F. (1996). *Aust. J. Chem.* **49**, 659–667.

[bb8] Charan, R. D., Schlingmann, G., Janso, J., Bernan, V., Feng, X. & Carter, G. T. (2004). *J. Nat. Prod.* **67**, 1431–1433. 10.1021/np040042r 15332871

[bb9] Evans, P. (2006). *Acta Cryst.* D**62**, 72–82. 10.1107/S0907444905036693 16369096

[bb10] Feling, R. H., Buchanan, G. O., Mincer, T. J., Kauffman, C. A., Jensen, P. R. & Fenical, W. (2003). *Angew. Chem. Int. Ed.* **42**, 355–357. 10.1002/anie.200390115 12548698

[bb11] Fenical, W. & Jensen, P. R. (2006). *Nat. Chem. Biol.* **2**, 666–673. 10.1038/nchembio841 17108984

[bb12] Fu, P. & MacMillan, J. B. (2015). *J. Nat. Prod.* **78**, 548–551. 10.1021/np500929z PMC4380196 25584783

[bb13] Fu, P., Wang, S., Hong, K., Li, X., Liu, P., Wang, Y. & Zhu, W. (2011). *J. Nat. Prod.* **74**, 1751–1756. 10.1021/np200258h 21770434

[bb14] Garden, S. J., Corrêa, M. B., Pinto, A. C., Wardell, J. L., Low, J. N. & Glidewell, C. (2007). *Acta Cryst.* C**63**, o234–o238. 10.1107/S0108270107008682 17413236

[bb15] Hua, G., Du, J., Slawin, A. M. Z. & Woollins, J. D. (2014). *J. Org. Chem.* **79**, 3876–3886. 10.1021/jo500316v 24678675

[bb16] Kwon, H. C., Kauffman, C. A., Jensen, P. R. & Fenical, W. (2006). *J. Am. Chem. Soc.* **128**, 1622–1632. 10.1021/ja0558948 16448135

[bb17] Lam, K. S. (2006). *Curr. Opin. Microbiol.* **9**, 245–251. 10.1016/j.mib.2006.03.004 16675289

[bb18] Luesch, H., Yoshida, W. Y., Moore, R. E., Paul, V. J. & Corbett, T. H. (2001). *J. Am. Chem. Soc.* **123**, 5418–5423. 10.1021/ja010453j 11389621

[bb19] Mague, J. T., Mohamed, S. K., Akkurt, M., Hassan, A. A. & Albayati, M. R. (2014). *Acta Cryst.* E**70**, o907–o908. 10.1107/S1600536814016298 PMC4186150 25309246

[bb20] MarXperts. (2015). *Automar*. MarXperts GmbH, D-22844 Norderstedt, Germany.

[bb21] Molinski, T. F., Dalisay, D. S., Lievens, S. L. & Saludes, J. P. (2009). *Nat. Rev. Drug Discov.* **8**, 69–85. 10.1038/nrd2487 19096380

[bb22] Moreno-Fuquen, R., Castillo, J. C., Becerra, D., Camargo, H. & Henao, J. A. (2015). *Acta Cryst.* E**71**, o882–o883. 10.1107/S2056989015019192 PMC4645012 26594578

[bb23] Nayak, S. K., Venugopala, K. N., Chopra, D., Govender, T., Kruger, H. G., Maguire, G. E. M. & Guru Row, T. N. (2009). *Acta Cryst.* E**65**, o2611–o2612. 10.1107/S1600536809039543 PMC2971060 21578228

[bb24] Parsons, S., Flack, H. D. & Wagner, T. (2013). *Acta Cryst.* B**69**, 249–259. 10.1107/S2052519213010014 PMC3661305 23719469

[bb25] Şen, F., Dinçer, M., Çukurovalı, A., Yılmaz, İ. (2013). *J. Mol. Struct.* **1046**, 1–8.

[bb26] Sheldrick, G. M. (2008). *Acta Cryst.* A**64**, 112–122. 10.1107/S0108767307043930 18156677

[bb27] Sheldrick, G. M. (2015). *Acta Cryst.* C**71**, 3–8.

[bb28] Shimanaka, K., Kinoshita, N., Iinuma, H., Hamada, M. & Takeuchi, T. (1994). *J. Antibiot.* **47**, 668–674. 10.7164/antibiotics.47.668 8040071

[bb29] Taori, K., Paul, V. J. & Luesch, H. (2008). *J. Am. Chem. Soc.* **130**, 1806–1807. 10.1021/ja7110064 18205365

[bb30] Yun, B. S., Hidaka, T., Furihata, K. & Seto, H. (1994). *J. Antibiot.* **47**, 510–514. 10.7164/antibiotics.47.510 8195055

[bb31] Zabriskie, T. M., Foster, M. P., Stout, T. Y., Clardy, J. & Ireland, C. M. (1990). *J. Am. Chem. Soc.* **112**, 8080–8084.

